# Dietary practice and nutritional status and the respective effect of pulses-based nutrition education among adolescent girls in Northwest Ethiopia: a cluster randomized controlled trial

**DOI:** 10.3389/fnut.2023.1102106

**Published:** 2023-09-25

**Authors:** Fantahun Ayenew Mekonnen, Gashaw Andargie Biks, Telake Azale, Netsanet Worku Mengistu

**Affiliations:** ^1^Department of Epidemiology and Biostatistics, Institute of Public Health, College of Medicine and Health Sciences, University of Gondar, Gondar, Ethiopia; ^2^Department of Health Systems and Policy, Institute of Public Health, College of Medicine and Health Sciences, University of Gondar, Gondar, Ethiopia; ^3^Department of Health Education and Behavioral Sciences, Institute of Public Health, College of Medicine and Health Sciences, University of Gondar, Gondar, Ethiopia; ^4^Department of Human Nutrition, Institute of Public Health, College of Medicine and Health Sciences, University of Gondar, Gondar, Ethiopia

**Keywords:** cluster randomized trial, nutrition education, adolescent girls, adolescents, pulses, legumes, thinness, underweight

## Abstract

**Background:**

Thinness and stunting are the most severe public health problems among adolescent girls in Ethiopia. An inadequate intake of protein-source foods is the most critical cause, mainly due to the non-affordability of animal-origin foods. However, research into what extent improving pulses-based food consumption could contribute to decreasing the magnitude of protein-energy undernutrition is limited.

**Objective:**

This trial aimed to evaluate the effectiveness of pulses-based nutrition education in reducing the proportion of thinness among adolescent girls.

**Methods:**

A two-arm cluster randomized controlled trial was conducted among adolescent girls in Northwest Ethiopia from December 2021 to June 2022. A total of 602 adolescent girls from four schools were enrolled in the trial. Schools were assigned to intervention and control groups using the stratified cluster randomization method. Pulses-based nutrition education was the intervention, whereas the usual dietary practice of adolescent girls was the comparator. The education was delivered over 4 weeks on a 45–60-min session per week basis. Thinness was the primary outcome of the trial, measured by anthropometry. An intention-to-treat analysis method was used. A log-binomial regression model was fitted to the data. Relative risk with the respective confidence interval and value of *p* was calculated. A value of *p* < 0.05 was used to declare statistical significance. Stata 16 software was used for the analysis.

**Results:**

About 89.37% of the participants in the intervention group and 92.36% in the control group completed the trial. The pulses-based nutrition education intervention did not show a significant difference in reducing the proportion of thinness among the participants in the intervention group compared to the participants in the control group even though a significant difference was observed in terms of the consumption of pulses-based food.

**Conclusion:**

The present trial was statistically non-significant in reducing thinness among adolescent girls. Similar studies that utilize objective methods for ascertaining pulses-based food consumption need to be conducted.

**Clinical trial registration**: https://pactr.samrc.ac.za/Search.aspx, the trial was registered in the Pan African Clinical Trials Registry (PACTR202111605102515) on November 12, 2021.

## Introduction

Adolescent girls’ protein undernutrition remains one of the most critical public health burdens in low- and middle-income countries (LMICs) (1–4). For instance, an analysis of worldwide trends in body mass index (BMI), underweight, overweight, and obesity from 1975 to 2016 showed the mean BMI estimates for youths aged 10–19 years old in South Asia, South-East Asia, East Africa, West Africa, and Central Africa were <20. According to the report, Ethiopia is among countries such as Niger, Senegal, India, Bangladesh, Myanmar, and Cambodia, where the lowest BMIs in the world were recorded (5). The Ethiopian Demographic and Health Survey (EDHS) also reported that 29% of adolescent girls in Ethiopia were underweight in 2016 (6). There are also similar recent studies in the country that reported underweight and stunting prevalences ranging from 13.6–29% (7–12) and 11.9–47.4% (9, 10, 12–15), respectively.

Although protein-energy undernutrition is known to cause significant morbidity and mortality among adolescent girls, its intergenerational consequences on their reproductive outcomes are the most serious. For instance, underweight adolescent girls are more likely to be underweight mothers, making them give birth to small babies (16). In turn, the low birth weight baby is more vulnerable to several risks of illness, death, and developmental problems (17, 18). These lead to poor educational achievement, school attendance, and concentration, perpetuating the cycle of undernutrition across generations and heavily affecting the productivity of a given nation (19, 20). However, adolescence is, fortunately, the second opportunity to correct the nutritional deficiencies experienced during early childhood, thereby breaking the intergenerational cycle (21).

Several factors are responsible for adolescent girls’ protein undernutrition. However, inadequate energy and protein intake is believed to be the most critical cause of teenage girls’ thinness and stunting in LMICs, mainly due to the inaccessibility of protein-source foods and poor knowledge and attitudes about nutrition. Specifically, animal-origin food intakes, such as meat, eggs, and milk, are low in most low-income countries, including Ethiopia, and these foods are not affordable for most of their populations (1, 8, 22–25). However, it is strongly recommended that people consume pulses rich in quality protein and minerals, such as iron, calcium, and zinc, as an alternative protein source food when accessing animal-origin foods is a chronic problem. Pulses such as chickpeas, lentils, peas, and broad beans are much more accessible and affordable in many low-income countries (26–28). Pulses are the most commonly produced crops in Ethiopia, which is among the top 10 pulses crop producer countries globally, and they are relatively more affordable than animal-origin foods for most Ethiopians (26, 29). However, studies, including our baseline survey, showed poor preparation and low consumption of pulses-based food due to the lack of knowledge about the benefits and preparation of pulses (30, 31). Therefore, this study evaluated the effectiveness of pulses-based nutrition education in reducing the proportion of thinness among adolescent girls in Northwest Ethiopia.

## Methods

### Trial design

A stratified cluster randomized controlled trial was conducted among adolescent girls. In the present trial, clusters were schools, and the study units were adolescent girls from the respective schools. The study had two groups, an intervention group (IG) and a control group (CG). A total of four clusters were identified and assigned to the intervention group and the control group using the stratified cluster randomization method. Adolescent girls from the respective schools were enrolled into the groups using a systematic random sampling method. In the present trial, the intervention was pulses-based nutrition education, while the comparator was the usual dietary practice of adolescent girls.

### Participants

A total of 602 adolescent girl participants, 301 for each arm, from four schools in Central Gondar Zone, Northwest Ethiopia, were enrolled in the trial. The participants were girls between 15 and 19 years old, attending grades 9–12. Only adolescent girls who were willing to participate were included in the trial. Those participants who could not communicate and planned to leave the study area/school before the study completion were excluded from the trial. On the other hand, schools found in areas where pulse crop production is prevalent were included. Whenever schools were considered close to each other, the one situated between those schools was excluded and then used as a buffering zone to avoid information contamination. Participant recruitment was conducted in the 1st week of December 2021.

### Study setting

This study was conducted among adolescent girls in the Central Gondar zone, located 726 km away from Addis Ababa, in northwest Ethiopia. The area has 15 districts and 29 schools serving about 46,340 students. There are two climatic conditions in the Central Gondar Zone, highland and lowland; thus, there are different states of pulse crop production. Pulse crop production is high in the northern and southern parts of the zone, while there is little in the eastern part, and rare in the western part. However, common in the north and the south, the pulses produced differ due to the difference in climatic conditions. The most prevalent pulse crop grown in the south is chickpea, while pea and broad bean are harvested in the north (communications with Central Gondar Zone Education and Agriculture offices).

### Intervention

The intervention was pulses-based nutrition education and the comparator was the usual dietary practices of adolescent girls. Adolescent girls in the intervention group received weekly lessons for 4 weeks on a one 45–60 min session per week basis.

The first session was about the definition and short and long-term consequences of undernutrition for adolescent girls. The second session overviewed the food groups’ sources and functions. The third and fourth sessions were about pulses such as broad beans, peas, chickpeas, and lentils, which are accessible in the study area and relatively affordable for the trial participants. To be more specific, the third session was about the recommended daily quantity of pulses intake and pulses processing (cleaning, washing, soaking, germination, and boiling). The benefits of further processing of pulses in terms of removing anti-nutrients and improving test and digestibility, mixing pulses with cereals to enhance the quality of their protein, and the different pulses-based recipes were also discussed in this session. The fourth session was a demonstration of selected pulses-based recipes.

The selection considered the relative ease of accessing the pulses and other inputs and the ease in terms of time and skill to prepare them. As a result, only pulses-based recipes, such as recipes for soup and pulses mixed with rice and vegetables, were demonstrated. Pulses and rice were the main components used in the demonstration, while vegetables, spices, and oil were included to enhance the flavor of the food. Broad beans were used for the demonstration conducted in the North, while chickpeas were used in the South. The investigators delivered the theoretical sessions while a chef conducted the demonstration. The maximum number of participants per training session for the theoretical sessions was 50, while all the participants in the intervention group attended the demonstration and were served the recipe at a time.

To enhance adherence to the nutrition education sessions, participants who were unwilling to participate after explaining the purpose, risks, and benefits of participating in the intervention were excluded. We also excluded participants who had planned to leave the school during the study duration. A nutrition education manual was developed and strictly followed.

The nutrition education manual was developed by reviewing the Food and Agriculture Organization’s (FAO) materials related to general dietary knowledge, attitude and practice assessment guidelines, and guidelines specific to pulse processing and pulses-based food preparation (26, 27, 32). Pender’s Health Promotion Model (HPM) manuals and procedures were also used (33–35). The manual was presented to experts, and their feedback was received and incorporated. The nutrition education was delivered from the 4th week of December 2021 to the 3rd week of January 2022.

### The outcome of the intervention

Thinness was the primary outcome of the current trial, while pulses-based food consumption and knowledge, beliefs, perceptions, and experiences about pulses-based food were the secondary outcomes. Thinness was categorized based on z-scores calculated using the WHO AnthroPlus software. Accordingly, adolescent girls with a body mass index-for-age (BMI-for-age) Z-scores of < − 2 were classified as thin (36, 37).

Pulses-based food consumption was dichotomized into ‘yes’ or ‘no’ levels and was based on 24-h dietary recall. The consumption was regarded as ‘yes’ when the participant reported consuming pulses-based food in the past 24 h in one or more of the following forms: roasted, boiled, germinated, or bread mixed with cereals and vegetables. The pulse quantity alone was a cup or a handful to an approximate amount of 100–120 g or a cup of cooked pulses consumed in the past 24 h (26, 28).

Furthermore, the knowledge, beliefs, perceptions, and experiences about pulses-based food were organized based on Pender’s Health Promotion Model domains (35). The domains relevant for this particular trial were: (1) commitment to prepare and consume pulses-based food, (2) pulses taste-related barriers to consuming pulses-based food, (3) self-efficacy beliefs to consume pulses-based food, (4) perceived benefit of consuming pulses-based food, (5) interpersonal influence to consume pulses-based food, and (6) knowledge/skill/accessibility-related barriers to consume pulses-based food. The domains were measured using 28 questions with a 5-point Likert scale. The domains were dichotomized as favorable and unfavorable using the sum of the middle value “3” as the cutoff point. Thus, when a participant’s items sum score for a given domain was greater than the sum score of the middle value of that domain, then that participant was categorized as having favorable behavior and if otherwise, as having unfavorable behavior.

Finally, the dietary diversity score was categorized as good if the trial participant reported consuming five or more of the 10 food groups. The food groups included cereals/roots/tubers, pulses, nuts and seeds, dairy food, meat and poultry, egg, green leafy vegetables, other vitamin A-rich fruits and vegetables, other vegetables, and other fruits. A food group was considered consumed in the past 24 h if the serving of food items in that group was one tablespoon size or about a fistful, which is equivalent to 15 g (38). Baseline data were collected in the 3rd week of December 2021, and end-line data were collected in the first week of June 2022. The time gap from the last nutrition education session to the post-intervention outcome measurement was 4 months and 1 week.

### Randomization

Schools were the units of randomization into intervention and control groups. Four schools were randomized using the stratified cluster randomization method. Stratification was based on climatic conditions, and thus two geographic areas were formed. Two schools were purposively selected from each geographic area stratum. The schools were chosen so that they were reasonably far apart. The two schools from each stratum were finally randomized into the intervention and control groups using an envelope. Therefore, each stratum/geographic area was made to have one intervention and one control school/cluster. Adolescent girls were then recruited using systematic random sampling from each school. Study participants from the intervention schools were the intervention recipients, and study participants from the control schools were left without the intervention and continued with their usual dietary practices.

### Allocation concealment and blinding

Consent and recruitment of the schools and individual study participants were conducted before the schools were randomized into intervention and control groups. Baseline data were collected from the study participants before the schools were randomized. The personnel facilitating the self-administered questionnaire and measuring the weight and height were also unaware of which participants or schools were part of the intervention group and which were not. One academic staff in the Department of Epidemiology and Biostatistics at the University of Gondar conducted the randomization procedure.

### Sample size estimation

The sample size was calculated using proportions of the outcome (BMI-for age) before and after the intervention based on an individually randomized trial. The previous study showed that the prevalence of thinness among adolescent girls was 13.6% before and 3% after the intervention (39). The sample size, considering an individually randomized trial by assuming 0.9 power and a 5% level of significance, was 274 in total, 137 for each group. After multiplying by a design effect of 2 and adding a 15% loss to follow-up, the final sample size was 602 participants, 301 for each arm.

### Data collection procedure

Baseline and end-line anthropometric data were collected using a digital weight scale and stadiometer. Data relating to dietary practice, including consumption of pulses-based food and knowledge, beliefs, perceptions, and experiences about pulses-based food, and sociodemographic variables were collected using a self-administered questionnaire.

Weight was measured using an electronic SECA scale after the participants removed their shoes and heavy clothing, and the measurement was recorded to the nearest 0.1 kg. Height was measured using a stadiometer with a sliding headpiece. The value was registered to the nearest 0.1 cm. The measurement was conducted after the participant removed her shoes and anything on her head, stood on the basal part of the stadiometer with feet together, the shoulders, buttocks, calves, and heels touched the vertical stand, and eyes in the Frankfort horizontal plane. The digital weight scale and the stadiometer were placed on a hard-flat table. All the procedures were aligned with the Food and Nutrition Technical Assistance (FANTA) 2018 anthropometry guide (40).

Data on the consumption of pulses-based food were collected using the past 24-h pulses-based food consumption history adopted from the 24-h recall dietary practice assessment approach. Thus, the questions were the FAO’s six close-ended questions, with ‘yes’ or ‘no’ options, followed by six probing questions (32). The probing questions were used to list the various forms of pulses-based food consumed at a particular point in time in the past 24 h. The questions had explicit instruction for the students that they should choose the ‘yes’ option if the pulses they consumed in the past 24 h were about the size of a handful or a cup, which is assumed to be equivalent to 100–120 g (26, 28). In addition, the pulses should be consumed after mixing with cereals.

Concerning data collection on the knowledge, beliefs, perceptions, and experiences about pulses-based food, a tool of 29 items with an acceptable item consistency (Cronbach’s alpha = 0.72) was used and organized based on Pender’s Health Promotion Model domains (35). The domains with the respective number of items were (1) commitment to prepare and consume pulses (5 items, e.g., − I am committed to preparing and eating pulses-based food in boiled forms mixed with cereals), (2) pulse taste-related barrier to consuming pulses (9 items, e.g., − I do not eat pulses food in boiled form since I do not like its taste), (3) self-efficacy beliefs to consume pulses (6 items, e.g., − I can plan to prepare and eat pulses in a germinated form mixed with cereals), (4) the perceived benefit of consuming pulses (2 items, e.g., −Eating pulses combined can help increase growth and development), (5) interpersonal influence to consuming pulses (4 items, e.g., − My friends encourage me to consume pulses foods), and (6) knowledge/skill/accessibility barriers to consuming pulses (3 items, e.g., − Since I do not know, I cannot prepare and consume pulses mixed with rice). The items were designed to be on a 5-point Likert scale. In addition to Pender’s Health Promotion Model (HPM) manual, other related guidelines were also used to develop the items (33–35).

Data related to general dietary practice focused on the details of dietary intake in the past 24 h. Six FAO’s dietary practice assessment close-ended questions with a ‘yes’ or ‘no’ response option were used. The questions were about any food or drink, except water consumed in the past 24 h at six different time points and between (early in the morning, late in the morning, noon, afternoon, evening, and before going to bed). Each close-ended question was followed by a probing question with adequate space to write details of what was eaten and drunk, provided that the answer for the close-ended question was ‘yes’. These dietary practice assessment questions had an explicit instruction for the participants that they should choose the ‘yes’ option if the food they consumed was roughly about the size of a tablespoon or a fistful, which is assumed to be equivalent to 15 g (32, 38, 41).

### Quality control

Several data quality assurance activities were undertaken at the different steps of the study. Participants were enrolled in the trial after being well introduced to the trial objective and how much time they could spend answering the questions. The data were collected whenever the participants had free classes, at break time, before the day’s class began, or after class ended, considering their preferences. When the filled questionnaire was returned, corrections for completeness, consistency, and accuracy was done on the spot before the student left the room.

There was an attempt to make pre-and post-test data collection contexts (timing and place) as similar as possible. The digital weight scale was regularly calibrated at a 10 kg weight. The variables were measured by trained personnel using an anthropometric data collection protocol, and the overall data collection was closely supervised. Finally, this report was prepared using the Consolidated Standards of Reporting Trials (CONSORT) statement for cluster randomized trials (42).

### Statistical analysis

Intention-to-treat was the method of analysis. The Chi-square test was used to examine the baseline socio-demographic characteristic differences between the intervention and control groups and examine the changes between the pre-test and post-test measurements of the primary and secondary outcome variables. A Log-binomial regression model was fitted to the data, and the relative risk with the corresponding confidence interval and value of *p* was then produced to determine the strength, precision, and significance of the effect of the intervention on the outcome variable. A value of *p* < 0.05 was considered statistically significant. Sensitivity analysis was also conducted to examine the influence of loss to follow-up, non-adherence, and confounding variables on the effect of the intervention on the outcome variable. Stata version 16 was used for data analysis.

### Sensitivity analysis

Sensitivity analysis was conducted to examine the influence of the loss to follow-up, non-adherence, and confounding variables on the effect of the intervention. The effect of the intervention, excluding those losses to follow-up, was compared to the effect of the intervention keeping losses to follow-up or imputing the missing anthropometric data. The lost data imputation method was multiple imputations by assuming missing at random (MAR). However, we assessed statistically whether the missingness was missing completely at random (MAR), missing at random (MAR), or missing not at random (MNAR). Accordingly, thinness and other variables at baseline were compared between non-responders and responders and between non-responders of the intervention and control groups.

The influence of non-adherence was assessed by comparing the effect of the intervention on the participants who attended the four training sessions with the participants enrolled in the trial. Similarly, the influence of the baseline characteristics was assessed by conducting a multivariable analysis. All the sensitivity analyses were conducted to examine if the deviations could have affected the conclusion of the trial.

## Results

### Participant flow diagram

**Figure 1:**
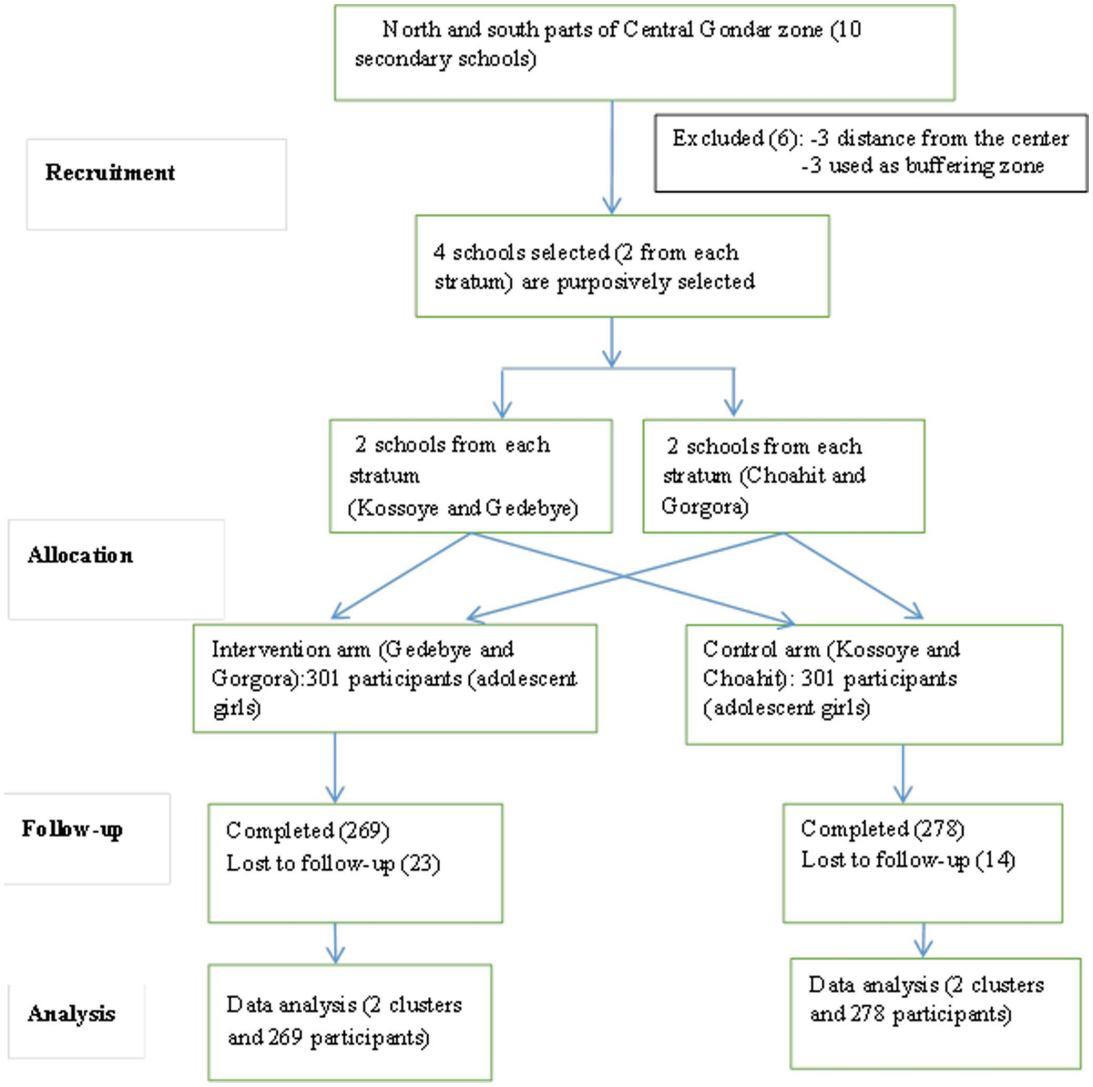
Flow diagram of pulses-based nutrition education compared to usual dietary practice among adolescent girls in Northwest Ethiopia, 2022.

A total of 602 participants, 301 from each arm, were enrolled in the study from the four schools, and 269 participants in the intervention arm and 278 participants in the control arm completed the trial, with 89.37 and 92.36% response rates, respectively. School dropout was the cause of the trial participants’ loss to follow-up (Figure 1).

### Baseline socio-demographic characteristics

Of the participants who completed the trial, most of the intervention participants, 82 (30.50%), were aged 18 years, and of the control participants, 68 (24.46%) were 17 years old, while the least frequent ages were 15 years old, 35 (13.00%), and 19 years old, 41 (14.75%), among intervention and control participants, respectively. In total, 28% of the intervention and 39.93% of the control participants were in grade 9, while 22.32% of the intervention and 14.75% of the control participants were in grade 12. The majority were rural dwellers, 181 (67.33%) of the intervention participants and 235 (84.53%) control participants. Concerning the participants’ history of school club participation, 189 (70.31%) of the participants in the intervention group and 218 (78.41%) of the participants in the control group reported that they had ever participated in the school club activity (Table 1).

**Table 1:** Baseline socio-demographic characteristics of adolescent girls in Northwest Ethiopia, 2022 (*n* = 547).

Characteristics	IG Freq (%)	CG Freq (%)	*χ*^2^ *p*-value
Age (years)			0.005
15	35 (13.00)	52 (18.70)	
16	48 (17.86)	63 (22.66)	
17	51 (18.97)	68 (24.46)	
18	82 (30.50)	54 (19.42)	
19	53 (19.72)	41 (14.75)	
Grade			0.003
9	77 (28.64)	111 (39.93)	
10	65 (24.18)	78 (28.06)	
11	67 (24.92)	48 (17.27)	
12	60 (22.32)	41 (14.75)	
Religion			0.05
Orthodox Christian	261 (97.09)	276 (99.28)	
Muslim	8 (2.98)	2 (0.72)	
Permanent residence			0.000
Rural	181 (67.33)	235 (84.53)	
Urban	88 (32.74)	43 (15.47)	
Temporary residence			0.000
Rural	101 (37.57)	148 (53.24)	
Urban	168 (62.50)	130 (46.76)	
School club participation			0.03
Yes	189 (70.31)	218 (78.41)	
No	80 (29.76)	60 (21.58)	

IG, intervention group; CG, control group; Freq, frequency.

### Dietary diversity and meal frequency

The dietary practice was similar between the intervention and the control groups at the baseline and end-line of the intervention across eight of the 10 food groups. There was a difference for other vegetables and other fruits at the baseline of the intervention and other fruits at the end-line of the intervention. Similarly, the dietary diversity score was different at the baseline but similar at the end-line of the intervention between the intervention and the control groups. Regarding meal frequency, no difference was observed between the intervention and control groups at both the baseline and end-line of the intervention (Table 2).

**Table 2:** Dietary diversity among adolescent girls in Northwest Ethiopia, 2022.

Food groups	Baseline			End-line		
	IG Freq (%)	CG Freq (%)	χ^2^ *p*-value	IG Freq (%)	CG Freq (%)	*χ*^2^ *p*-value
Cereals and roots	269 (100.00)	276 (99.28)	0.163	267 (99.26)	278	0.15
Pulses	238 (88.48)	245 (88.13)	0.9	259 (96.28)	263 (94.60)	0.35
Nuts and seeds	0 (0.00)	0 (0.00)	–	0 (0.00)	0 (0.00)	–
Diary food	25 (9.29)	40 (14.39)	0.08	11 (4.10)	11 (3.96)	0.9
Meat, poultry, and fish	107 (39.78)	104 (37.41)	0.57	43 (16.04)	52 (18.71)	0.41
Egg	14 (5.22)	18 (6.47)	0.534	4 (1.53)	6 (2.21)	0.56
Dark green leafy vegetables	4 (1.50)	2 (0.72)	0.391	1 (0.38)	1 (0.36)	0.98
Other vitamin A-rich fruits and vegetables	2 (0.74)	1 (0.36)	0.550	1 (0.40)	0 (0.00)	0.3
Other vegetables	4 (1.5)	0 (0.00)	0.044	2 (0.75)	1 (0.36)	0.54
Other fruits	13 (4.8)	37 (13.36)	0.001	13 (4.8)	37 (13.36)	0.001
Dietary diversity score	24 (8.92)	42 (15.11)	0.03	8 (3.00)	9 (3.24)	0.86
Meal frequency (≥3)	249 (92.57)	261 (93.88)	0.539	245 (91.08)	254 (91.36)	0.91

IG, intervention group; CG, control group; Freq, frequency.

### Knowledge, beliefs, perceptions, and experiences about pulses-based food

All six domains were not significantly different between the intervention and the control groups at the baseline of the intervention. However, one of the six domains showed a statistically significant difference between the groups at the end-line of the intervention. That is, interpersonal influence to consume pulses-based foods changed from a statistically insignificant difference (value of *p* = 0.49) at baseline to a statistically significant difference (value of *p* = 0.02) at the end-line between the IG and CG (Table 3).

**Table 3:** Knowledge, beliefs, perceptions, and experiences about pulses foods among adolescent girls in Northwest Ethiopia, 2022.

Barriers and facilitators	Baseline			End-line		
	IG Freq (%)	CG Freq (%)	*χ*^2^ *p*-value	IG Freq (%)	CG Freq (%)	*χ*^2^ p-value
Commitment to prepare and consume pulses	153 (56.88)	156 (56.12)	0.86	208 (77.32)	201 (72.30)	0.18
Pulses taste-related barrier to consume pulses	44 (16.36)	59 (21.22)	0.15	45 (16.73)	57 (20.50)	0.26
Self-efficacy beliefs to consume pulses	197 (73.23)	185 (66.55)	0.09	218 (81.04)	231 (83.09)	0.53
Perceived benefit of consuming pulses	190 (70.63)	192 (69.06)	0.69	239 (88.85)	240 (89.22)	0.37
Interpersonal influence to consuming pulses	93 (34.57)	104 (37.41)	0.49	101 (37.55)	79 (28.42)	0.02
Knowledge/skill/and accessibility barriers to consume pulses	70 (26.02)	58 (20.86)	0.15	70 (26.02)	59 (21.22)	0.15

IG, intervention group; CG, control group; Freq, frequency.

### Pulses-based food consumption

There was no statistically significant difference between the intervention group and the control group in terms of the consumption of five of the six pulses-based food forms, which included roasted, boiled, germinated, soup, bread, and mixed with cereals and/or vegetables at the baseline of the intervention in the past 24 h. The form of pulses-based food that showed a significant difference at baseline among the groups was bread (*p* = 0.008). Conversely, a statistically significant difference was observed between the intervention group and the control group at the end-line of the intervention in four of six pulses-based food forms. The changes in the Chi-square *p*-values of 0.14–0.001, 0.57–0.01, 0.008–0.0001, and 0.68–0.007 were for the boiled, germinated, bread, and vegetable forms, respectively. The pulses-based foods that remained insignificant were the soup and roasted forms. Regarding the overall pulses-based food consumption, a statistically significant difference was observed between the intervention and the control groups both at the baseline (*p* = 0.004) and end-line (*p* = 0.000) of the intervention (Table 4).

**Table 4:** Pulses-based food consumption among adolescent girls in Northwest Ethiopia, 2022.

Pulses-based food	Baseline			End-line		
	IG Freq (%)	CG Freq (%)	*χ*^2^ *p*-value	IG Freq (%)	CG Freq (%)	*χ*^2^ *p*-value
Boiled form	21 (7.81)	13 (4.74)	0.14	27 (10.04)	8 (2.89)	0.001
Germinated form	11 (4.09)	14 (5.11)	0.57	16 (5.95)	5 (1.81)	0.012
Bread form	32 (11.90)	15 (5.49)	0.008	35 (13.01)	6 (2.17)	0.000
Soup form	12 (4.48)	5 (1.83)	0.078	5 (1.86)	3 (1.09)	0.454
Mixed with vegetable	16 (5.95)	8 (2.88)	0.08	19 (7.06)	2 (0.720)	0.000
Overall pulses consumption	68	43	0.004	69	20	0.000

IG, intervention group; CG, control group; Freq, frequency.

### The effect of pulses-based nutrition education in reducing thinness

The primary analysis showed that the pulses-based nutrition education intervention did not significantly reduce thinness among the intervention group participants compared to the control group [CRR;95%CI: 0.56 (0.21, 1.50)]. The conclusion remained the same after the baseline outcome variable, thinness, was included in the analysis model [ARR; 95%CI: 1.32 (0.60, 2.92)] (Table 5).

**Table 5:** The effect of school-based pulses-based nutrition education in reducing thinness among adolescent girls in Northwest Ethiopia, 2022 (*n* = 547).

Characteristics	Thinness		CRR (95%CI)	ARR (95%CI)
	Yes	no		
Baseline thinness				
Yes	13	26	48 (14.48, 123.70)	33.39 (11.30, 98.72)
No	4	504	1	
Intervention				
Nutrition education	6	263	0.56 (0.21, 1.50)	1.32 (0.60, 2.92)
Control	11	267	1	

CRR, crude relative risk; ARR, adjusted relative risk.

### Sensitivity analysis

The sensitivity analysis revealed that the loss to follow-up and non-adherence did not result in a considerable change in the effect sizes of the intervention, as witnessed by the comparison of the effect sizes obtained from the per-protocol and intention-to-treat analyses. In addition, the conclusion regarding the effect of the intervention remained the same between the primary analysis and the analysis adjusted by baseline characteristics, including the baseline outcome variable (Table 5).

## Discussion

The present trial aimed to examine the effectiveness of pulses-based nutrition education in reducing the proportion of thinness among adolescent girls in Northwest Ethiopia. The trial revealed that the intervention was effective neither in the primary analysis [CRR; 95%CI: 0.56 (0.21, 1.50)] nor in the analysis adjusted by the baseline characteristics, including thinness at baseline, [ARR; 95%CI: 1.32 (0.60, 2.92)], in reducing the proportion of thinness among adolescent girls. The finding remained the same after the sensitivity analysis or when considering the influence of intervention adherence and loss to follow-up on the effect of the intervention. This finding is different from the findings of trials conducted among adolescent girls (44) and breastfeeding children (48–50) in the South Nations Nationalities and People’s Regional State (SNNPR), Ethiopia, which reported a significant reduction in the proportion of those underweight among the participants who received the pulses-based nutrition education as compared to those participants who did not receive the education. However, this finding is similar to the above previous findings (44, 48–50) in terms of the consumption of pulses-based food in that a statistically significant difference was observed in the consumption of the pulses-based food from the baseline to the end-line of the intervention between the participants in the intervention group and the participants in the control group. More specifically, the change in the consumption of pulses-based food in the current trial was a tiny increase among the participants in the intervention group, while there was a decrease among the participants in the control group from baseline to the end-line of the intervention. The deviation is because the baseline data were collected in December, one of the months in the harvesting season, while the end-line data were collected in June, one of the months in the non-harvesting season. In Ethiopia, June is one of the non-harvesting months in which most of the population in the area enters into a state of food stress and nutrition depletion (51, 52).

Therefore, the difference in the effectiveness of the intervention between the present study and the previous studies might be attributed to the difference in the data collection dates. In addition, the failure to account for the baseline variability in the analysis by the previous studies might, in part, have contributed to the difference. However, both the present and the previous studies applied objective assessment of the consumption of pulses-based food.

The limitation of the present trial is that data about the consumption of pulses-based food were collected by self-report, which might have resulted in an overestimation of the pulses-based food consumption proportion among the participants in the intervention group compared to the participants in the control group. However, the trial has the following strengths: an effort was made to make the trial participants represent different geographic areas, and thinness and other baseline characteristics were accounted for in the analysis.

## Conclusion

The present study revealed that pulses-based nutrition education intervention did not significantly reduce the proportion of thinness among adolescent girls in Northwest Ethiopia. However, this non-significant reduction of thinness is contrary to the favorable behavior observed toward the consumption of pulses-based food among the intervention group compared to the control group. Thus, a similar study using an objective measure of the consumption of pulses-based food needs to be conducted in the future. The future trial should not underestimate the influence that study participants’ baseline characteristics could have on the effect of the intervention.

## Data availability statement

The original contributions presented in the study are included in the article/supplementary material, further inquiries can be directed to the corresponding author.

## Ethics statement

The studies involving human participants were reviewed and approved by Institutional Review Board of University of Gondar. Written informed consent to participate in this study was provided by the participants’ legal guardian/next of kin.

## Author contributions

FM: conception, formulation of aims, data acquisition, analysis and interpretation of data, drafting and approval of article for publication and agreement to be accountable for all aspects of the work. GB, TA, and NM: formulation of aims and interpretation of data, revising and approval of article for publication and agreement to be accountable for all aspects of the work. All authors read and approved the final manuscript and met the ICMJE criteria for authorship.

## Funding

This protocol has received financial support from the University of Gondar (Grant no. 587/2020).

## Conflict of interest

The authors declare that the research was conducted in the absence of any commercial or financial relationships that could be construed as a potential conflict of interest.

## Publisher’s note

All claims expressed in this article are solely those of the authors and do not necessarily represent those of their affiliated organizations, or those of the publisher, the editors and the reviewers. Any product that may be evaluated in this article, or claim that may be made by its manufacturer, is not guaranteed or endorsed by the publisher.

## Abbreviations

BMI: Body mass index
BAZ: Body mass index-for-age z-score
CONSORT: Consolidated standards of reporting trials
EDHS: Ethiopian demographic and health survey
FAO: Food and agriculture organization
HAZ: Height-for-age z-score
HPM: Health promotion model
CRT: Cluster randomized trial
IG: Intervention group
CG: Control group
LMICs: Low and middle-income countries
PHPM: Pender’s health promotion model
